# Cost-effectiveness of screening with contrast enhanced magnetic resonance imaging *vs* X-ray mammography of women at a high familial risk of breast cancer

**DOI:** 10.1038/sj.bjc.6603356

**Published:** 2006-10-03

**Authors:** I Griebsch, J Brown, C Boggis, A Dixon, M Dixon, D Easton, R Eeles, D G Evans, F J Gilbert, J Hawnaur, P Kessar, S R Lakhani, S M Moss, A Nerurkar, A R Padhani, L J Pointon, J Potterton, D Thompson, L W Turnbull, L G Walker, R Warren, M O Leach

**Affiliations:** 1 MRC Health Services Research Collaboration, Department of Social Medicine, University of Bristol, Bristol, UK; 2 Nightingale Centre, Withington Hospital, Manchester, UK; 3 Addenbrooke's Hospital, Cambridge, UK; 4 Western General Hospital, Edinburgh, UK; 5 CRC Genetic Epidemiology Unit, Cambridge, UK; 6 MARIBS Study Office, Section Magnetic Resonance, The Institute of Cancer Research & the Royal Marsden NHS Trust, Downs Road, Sutton, Sussey SM2 5PT, UK; 7 Regional Genetics Service, Manchester, UK; 8 Department of Radiology, University of Aberdeen, Aberdeen, UK; 9 Department of Clinical Radiology, Manchester Royal Infirmary, Manchester, UK; 10 Discipline of Molecular & Cellular Pathology, School of Medicine, University of Queensland Mayne Medical School, Australia; 11 The Royal Marsden NHS Trust, London, UK; 12 The Paul Strickland Scanner Centre, Mount Vernon Hospital, Middlesex, UK; 13 MRI Unit, Royal Victoria Infirmary, Newcastle-upon-Tyne, UK; 14 Centre for Magnetic Resonance Investigations, Hull Royal Infirmary, Hull, UK; 15 Institute of Rehabilitation, University of Hull, Hull, UK

**Keywords:** cost-effectiveness analysis, breast MRI, screening, BRCA1 and BRCA2, breast cancer, high risk

## Abstract

Contrast enhanced magnetic resonance imaging (CE MRI) is the most sensitive tool for screening women who are at high familial risk of breast cancer. Our aim in this study was to assess the cost-effectiveness of X-ray mammography (XRM), CE MRI or both strategies combined. In total, 649 women were enrolled in the MARIBS study and screened with both CE MRI and mammography resulting in 1881 screens and 1–7 individual annual screening events. Women aged 35–49 years at high risk of breast cancer, either because they have a strong family history of breast cancer or are tested carriers of a *BRCA1*, *BRCA2* or *TP53* mutation or are at a 50% risk of having inherited such a mutation, were recruited from 22 centres and offered annual MRI and XRM for between 2 and 7 years. Information on the number and type of further investigations was collected and specifically calculated unit costs were used to calculate the incremental cost per cancer detected. The numbers of cancer detected was 13 for mammography, 27 for CE MRI and 33 for mammography and CE MRI combined. In the subgroup of *BRCA1* (*BRCA2*) mutation carriers or of women having a first degree relative with a mutation in *BRCA1* (*BRCA2*) corresponding numbers were 3 (6), 12 (7) and 12 (11), respectively. For all women, the incremental cost per cancer detected with CE MRI and mammography combined was £28 284 compared to mammography. When only *BRCA1* or the *BRCA2* groups were considered, this cost would be reduced to £11 731 (CE MRI *vs* mammography) and £15 302 (CE MRI and mammography *vs* mammography). Results were most sensitive to the unit cost estimate for a CE MRI screening test. Contrast-enhanced MRI might be a cost-effective screening modality for women at high risk, particularly for the *BRCA1* and *BRCA2* subgroups. Further work is needed to assess the impact of screening on mortality and health-related quality of life.

Women with a known family history of breast and/or ovarian cancer or mutations in *BRCA1* and *BRCA2* have a higher lifetime risk of breast cancer than the general population with tumours often occurring at a young age and more often being of a high grade. Several prevention strategies for these women have been identified including bilateral prophylactic mastectomy, chemoprevention and screening with annual mammography (Liberman, 2004; McIntosh *et al*, 2004).

Current guidance in the UK recommends that all women aged 40–49 years with a moderate risk (lifetime risk >17%) should be offered annual mammographic surveillance (McIntosh *et al*, 2004). There is, however, some concern about the poor sensitivity of mammography due to mammographically dense breast tissue in younger, premenopausal women and tumours resulting from gene mutations may potentially have a more aggressive phenotype (Liberman, 2004).

Over the last decade, magnetic resonance imaging (MRI) has emerged as a potential investigation for the detection and diagnosis of breast cancer and, unlike mammography, it is not affected by breast density. This has prompted a number of investigators to evaluate the feasibility of MRI in a screening context.

One of these studies is the UK national study for magnetic resonance imaging screening of women at high familiar risk of breast cancer (MARIBS) that was set up to compare the sensitivity and specificity of contrast-enhanced magnetic resonance imaging (CE MRI) with two-view mammography (Leach *et al*, 2005). This study and other prospective screening studies (Kriege *et al*, 2004; Warner *et al*, 2004; Kuhl *et al*, 2005) consistently showed that CE-MRI is significantly more sensitive than X-ray mammography (XRM) in a high-risk population.

There is some concern, however, that the reported lower specificity of CE MRI compared to mammography in three of these studies (Kriege *et al*, 2004; Warner *et al*, 2004; Leach *et al*, 2005) will require more short-term follow-up and additional costly investigations. Moreover, CE MRI is more sparsely available, requires more highly trained personnel, costly facilities and consumables, most notably the contrast medium. Therefore, evidence is needed to decide whether the potential benefits justify the additional costs required for implementing CE MRI in a surveillance programme for women at high familial risk of breast cancer.

Using data from the MARIBS study, the objective of this paper was to evaluate the cost-effectiveness of mammography alone (XRM), CE MRI alone, or mammography (XRM) in combination with CE MRI.

## MATERIALS AND METHODS

### Overview

The perspective of this economic analysis is that of the UK National Health Service, and all costs are reported in 2003 sterling prices including VAT. We used additional cancer detected as the measure of effectiveness corresponding to the clinical study (Leach *et al*, 2005).

### Clinical study

Full details of the design and the results of the clinical study have been published elsewhere (Leach *et al*, 2005). Briefly, the aim of this study was to compare the diagnostic accuracy of CE MRI and XRM. Women aged 35–49 years at high genetic risk of breast cancer (>0.9% per annum) were recruited between 1997 and 2004 from 22 centres in the UK when they fulfilled one the following entry criteria: tested carriers of a deleterious *BRCA1* or *BRCA2* or *TP53* mutation, first degree relative of someone with a *BRCA1* or *BRCA2* or *TP53* mutation or a strong family history of breast or ovarian cancer (Brown *et al*, 2000b). All participants were offered annual screening with both CE MRI and mammography. Mammography was performed according to the standards of the National Health Service Breast Screening Programme (NHSBSP). This was usually using two-view, but in a minority (7%) only one-view was taken (mediolateral oblique only). Screening CE MRI was undertaken utilising a specified protocol (Warren *et al*, 2005) and gadopentetate dimeglumine (Magnevist®, Schering Healthcare, UK) as contrast medium (bolus intravenous injection of 0.2 mmol kg^−1^ body weight). Both mammography and MRI were independently double reported (taking the most conservative score). True cancer status was ascertained by pathology (when a biopsy was taken) or by the absence or presence of interval cancers in the year after the screening event. In correspondence with the clinical analysis presented in the main study findings (Leach *et al*, 2005), the economic analysis is based on the 649 women who were both screened with CE MRI and mammography resulting in 1881 screens (1–7 screens over the 7 years of annual follow-up).

### Number of recalls

In the clinical study, a women was recalled for further assessment after a query was raised by a screening event on either or both modalities and a review of the results of both screening tests was performed by the supervising radiologist as an integrated whole. A radiologist and the subject's clinically responsible physician then made the choice of diagnostic pathway.

For the cost-effectiveness analysis it was, however, necessary to determine the number of women who would have been recalled for further assessment if the screening technology had been either mammography alone or CE MRI alone or both. We assumed that a recalled woman in the study would have been recalled on the basis of mammography alone if the first and second mammography reading was abnormal regardless of the CE MRI finding or if either the first or second mammography reading was abnormal and the CE MRI was normal. In the case of discordant mammography readings together with one and/or two abnormal CE MRI readings, it was not clear whether the woman would have been recalled on the basis of mammography alone because it is usual practice in the UK breast screening programme to resolve such cases by consensus or arbitration. In these cases, an additional radiologist who was blinded to the CE MRI results undertook a third reading of the mammograms in order to simulate these conditions. We considered women recalled on the basis of mammography alone when two of the three readers reported an abnormal mammography finding. We assumed that a recall on the basis of the CE MRI alone would have taken place if the first and/or second reading of the CE MRI were abnormal regardless of the mammography findings because, in practice, abnormal findings from either MRI reader were acted on. These assumptions are summarised in Box 1.

### Measurement of resource use

We estimated the cost associated with the screening itself and the costs associated with further investigations that were necessary to establish the final diagnosis for each recalled woman. Information on the type and number of all further investigations (including further mammograms, supplementary MRI screening studies, ultrasound, fine needle aspiration cytology, core biopsy, MRI-guided biopsy and surgical biopsy) was recorded within the MARIBS study.

### Unit cost

The cost associated with the screening tests and further investigations was estimated using information from five participating centres that screened the majority of the patients enrolled in the study, supplemented by data on unit costs from published national sources (Department of Health, 2005). Unit costs at 2003/2004 prices were based on the resources used in the study, the actual annual throughput of patients and the requirements of the research protocol (Brown *et al*, 2000a, 2000b). We collected information on staffing levels and timings for each phase of the screening and diagnostic tests, consumables, equipment and maintenance costs, capital charges and overheads. We then estimated the annual cost of equipment using lifetime estimates provided by the staff at the centres and a discount rate of 3.5% (NICE, 2004). The price for the contrast medium was based on the average weight of the women enrolled in the study, assuming no shared use of bottles. Staff time was costed assuming the midpoint of the annual salary scale plus oncosts (employer's National insurance and pension contributions) for each member of staff and published information on staff hours (Curtis and Netten, 2004).

Nevertheless, the unit cost associated with the research based CE MRI screening procedure is likely to represent an overestimate of the costs incurred in routine clinical practice because of the specific requirements of the research protocol used in the MARIBS study (Brown *et al*, 2000b). Improved CE MRI protocols for routine clinical practice will require less time for the MRI examination and subsequent analysis than dictated by the research protocol, which was designed to collect additional information to determine which features of a range of potential measurements provided the most sensitive and specific assessment of breast cancer (Brown *et al*, 2000b). Other cost reducing factors in routine practice are lower MRI equipment costs (compared with the highly specified systems purchased by research centres), more experienced radiologists working in breast CE MRI (Warren *et al*, 2005) and the possible introduction of generic formulations of Gd-DTPA coupled with potential lower dose than <0.2 mmol per kg (Knopp *et al*, 2003). We therefore estimated alternative unit cost based on the following assumptions:

- Equipment costs of £700 000;
- reduction of examination time by 36% (30 min instead of 47 min);
- reduction of time for analysis by 50% (15 min instead of 30 min);
- 50% reduction for cost of contrast medium (due to generic formulation of Gd-DTPA or a lower dose of 0.1 mmol kg^−1^ body weight);
- 5000 annual examinations per MRI machine (assuming 10 h running time of a scanner and 30 min duration per examination).

All other resource use estimates were taken as means from the five centres that participated in our costing survey. This cost estimate is used in the base case analysis. The other CE MRI unit cost estimate, based on the requirements of the research protocol, is used in sensitivity analyses. A breakdown of these unit costs associated with these different CE MRI factors is presented in Table 2.

### Statistical analyses

We used STATA (version 8.0) for general statistical analyses and Markov Chain Monte Carlo (MCMC) as implemented in the freely available software WinBUGS (Spiegelhalter *et al*, 2000) for the uncertainty analysis. Given that costs were incurred in the first year of the analysis, costs and outcomes were not discounted. Cost-effectiveness analysis was undertaken to relate differential mean costs and the number of cancers detected associated with mammography alone, CE MRI alone and a combined strategy, with incremental cost-effectiveness ratios (ICERs) calculated, as appropriate.

To account for uncertainty due to sampling variation, we used the net-benefit approach implemented in the statistical model depicted in equation (1). We plotted cost-effectiveness acceptability curves (Fenwick *et al*, 2001) showing the probability of one screening strategy being cost-effective compared to the two alternative screening strategies for different levels that a decision maker is willing to pay for an additional cancer detected. The probability that either mammography or CE MRI or mammography and CE MRI combined detected a cancer; the probability of recall was modelled using multinomial distributions. The cost associated with further investigations was modelled using a lognormal distribution. All parameters were given vague priors in the absence of prior information or expert opinion. The impact of different distributional assumptions (e.g. gamma-distributions) for the cost data was tested in sensitivity analyses (Nixon and Thompson, 2004). The results were based on a sample of 40 000 runs after a burn in period of 10 000 runs had been discarded (after assessing convergence with the Brooks Gelman Rubin diagnostic in WinBUGS).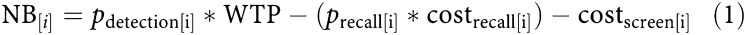 where NB_[i]_ is the net benefit; WTP the decision maker's willingness to pay per cancer detected; *p*_detection[i]_ the probability of a cancer being detected; *p*_recall[i]_ the probability for a recall; cost_recall[i]_ the cost associated with further investigations; cost_screen[i]_ the unit cost of screening test and index [i] indicates screening modality.

### Sensitivity analyses

We also analysed the cost-effectiveness of screening for women with a *BRCA1* (*BRCA2*) mutation or with a relative having a mutation in *BRCA1* (*BRCA2*) separately. In addition, we conducted several one-way sensitivity analyses to assess the impact of different unit cost assumptions (particularly CE MRI) on the incremental cost per cancer detected.

### Role of the funding source

The investigators were responsible for the study design, data collection, data analysis, data interpretation and writing the report, independently of all funding sources.

## RESULTS

### Cost of screening tests

Table 1 presents a summary of all unit costs. The cost of screening mammography based on the five centres (£33.50) is remarkably similar to the national average costs (£31.67; IQR £26.44–£39.65) reported as NHS references costs (Department of Health, 2005) for general mammography. The cost of a CE MRI screening procedure is £249.6 (clinical practice setting), and £405.1 (research setting) and thus approximately 7.5 and 12 higher than mammography. Table 2 shows a breakdown of cost associated with CE MRI. Key components are the costs for the contrast medium (26%); cost associated with staff (29%) and overhead/capital costs (18%).

### Recalls and health outcomes

In the main study, 279 women were recalled for further investigations. Of those, 26 women were recalled with one or two abnormal CE MRI readings and with discordant mammography readings. As described in the Materials and methods section, additional reporting undertaken by a third radiologist who was blinded to both previous CE MRI and mammography reports resulted in 15 normal and six abnormal results. In five cases, the mammograms could not be retrieved from individual screening centres. In these cases, we assumed that a woman would not have been recalled on the basis of mammography alone, but tested this assumption in a sensitivity analysis. Based on these assumptions, 20 fewer recalls would have been made under a mammography only scenario with one cancer being missed (see Table 3 for a comparison with the results of the clinical study (Leach *et al*, 2005)). The resulting recall rates for the screening modalities were 2.9% for mammography, 10.7% for CE MRI and 12.7 for both modalities combined. The number of cancers detected by mammography was 13 (0.00691 per screen), by CE MRI 27 (0.01435 per screen) and 33 (0.01754 per screen) by mammography and CE MRI combined (Table 5). For the *BRCA1* (*BRCA2)* group, the number of cancers detected was three out of 13 (6 out of 12), 12 out of 13 (7 out of 12) and 12 out of 13 (11 out of 12) by mammography, CE MRI and CE MRI and mammography combined respectively. Forty recalls were not justified by either the CE MRI or mammography scores, and were thus purely on the basis of the reader's judgement, reflecting a cautious approach in these high-risk individuals. Recall rates were higher in the first study year compared to subsequent years in all three groups (mammography 4.1 *vs* 2.2%; CE MRI 12.3 *vs* 9.9%; mammography and CE MRI combined 15.2 *vs* 11.4%).

### Resource use and costs

Table 4 shows the number of further investigations for all recalled women. Both, CE MRI and mammography and CE MRI combined would have resulted in a significant number of further investigations. The highest mean cost associated with further investigations resulted from CE MRI (£506.4) compared to £282.8 for mammography and £399.5 for mammography and CE MRI combined (Table 5). When the additional seven MRI studies were taken into account in the mammography group, the recall costs would have been £321.6 for this group. The mean total costs per screen were £41.7 for mammography, £304.0 for CE MRI and £342.4 for mammography and CE MRI combined. When considering only the *BRCA1* (*BRCA2*) group, the total mean costs per women screened were £43.7 (£55.7) for mammography, £323.0 (£317.5) for CE MRI and £361.2 (£369.3) for mammography and CE MRI combined.

### Cost-effectiveness

Cost-effectiveness is summarised in Table 5. These results suggest that the additional cost per additional cancer detected is £28 284 for mammography and CE MRI combined compared with mammography, after excluding CE MRI for extended dominance (ICER is higher compared to the next, more effective alternative (Drummond *et al*, 2005)). Given that the addition of mammography to CE MRI did not result in any additional cancer detected in the *BRCA1* group, the cost-effectiveness could only be computed for the comparison of CE MRI to mammography alone. In this subgroup, the additional cost per additional cancer detected by CE MRI would be £11 731. For the *BRCA2* group, the cost per additional cancer detected equates to £15 302 for CE MRI and mammography combined after excluding CE MRI for extended dominance.

### Addressing uncertainty

**Figure 1:**
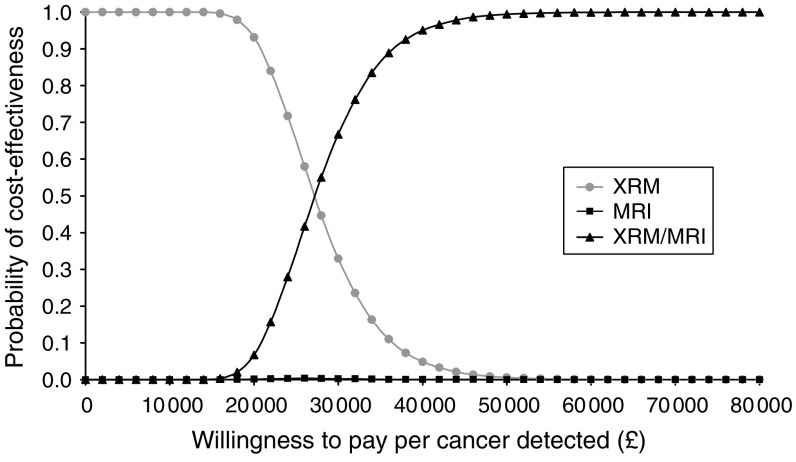
Cost-effectiveness acceptability curve (all women). Curve shows the probability that a screening modality is cost-effective for a range of decision makers' maximum willingness to pay per cancer detected.

Uncertainty around these estimates is represented by cost-effectiveness acceptability curves, which show the probability that one of the three screening combinations/options is cost-effective compared to a maximum willingness to pay that decision makers might have for these health outcomes (Figures 1 (for all women), 2 and 3 (for the *BRCA1* and *BRCA2* groups only)). For all women, the probability of CE MRI and mammography combined would be cost-effective is 0.07 for a decision maker's willingness to pay of £20 000 per cancer detected, or 0.67 for £30 000 per cancer detected.

### Sensitivity analyses

**Figure 4:**
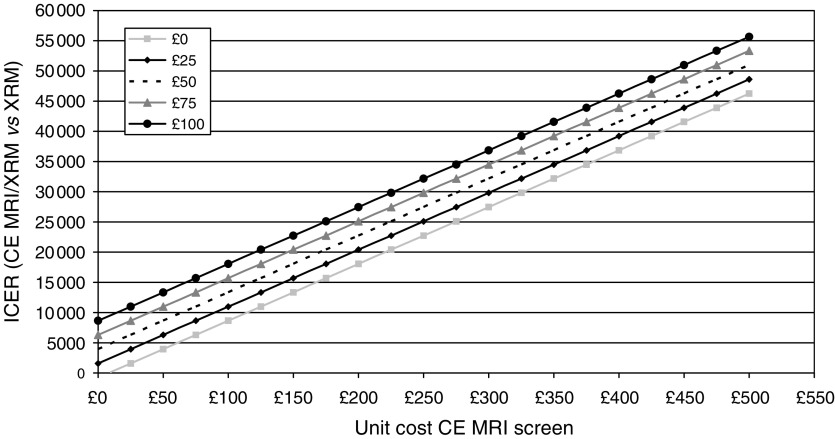
Sensitivity analyses: additional costs per cancer detected depending on the unit costs of screening CE MRI and cost associated with further investigations. ICER=incremental cost-effectiveness ratio (additional cost per additional cancer detected), lines represent different costs associated with further investigations.

When gamma-distributions were used to model costs instead of lognormal distributions, the corresponding probabilities were 0.06 and 0.65 respectively. For the *BRCA1* (*BRCA2*) group, the probability that CE MRI and mammography combined would be cost-effective would be 0.57 (0.82) and 0.71 (0.96) for a decision maker's willingness to pay per cancer detected of £20 000 and £30 000 respectively. Again, the use of gamma-distributions to model the costs associated with further investigations did not substantially alter our conclusions. The cost-effectiveness of CE MRI screening is most sensitive to the costs associated with the CE MRI screening test. Figure 4 shows the dependency of the incremental costs per cancer detected at different CE MRI costs and different levels of costs associated with further investigations to obtain the final diagnosis. As pointed out earlier, particularly lower recall rates and a reduced frequency of additional CE MRI examinations will result in lower costs to establish the final diagnosis. The incremental costs per cancer detected increased to £44 564 for all women, £19 068 for the *BRCA1* group and £22 890 for the *BRCA2* group when we used the unit costs of CE MRI based on the research setting (£405.1). The cost-effectiveness of CE MRI alone or in combination with mammography improves when only the prevalent screen is considered (Table 6).

## DISCUSSION

The findings of our analysis suggest that the addition of CE MRI to a surveillance programme for women at high familial risk for breast cancer might be a cost-effective health care intervention. This is particularly true for the *BRCA1* and *BRCA2* groups, which suggested that the incremental cost per detected cancer with CE MRI (combined with mammography or with CE MRI alone) is £11 800 and £15 300 respectively when compared to mammography alone.

Our results were most sensitive to the estimate of unit cost for one CE MRI screening examination. A number of factors support our assumptions underlying the lower unit cost estimate associated with the CE MRI screening tests used in the base case analysis. Firstly, adapted CE MRI protocols might be developed that require less time for the MRI examination and subsequent analysis than dictated by the research protocol (Warren *et al*, 2005) used in our pragmatic cohort study. Specific analysis software implemented on the MRI system promises to make analysis even easier. Secondly, due to the rapidly evolving developments of MRI technology, further reductions in equipment costs are likely to result in lower equipment costs per screening examination. Thirdly, there are likely to be cost reductions with regard to the contrast medium: a lower dose (0.1 mmol kg^−1^ body weight) of Gd-DTPA has been shown to be similarly effective (Knopp *et al*, 2003) compared to 0.2 mmol kg^−1^ used in our study and in addition, the expected availability of a generic formulation. Further cost reductions can be expected due to the increasing experience of radiologists with this new technique, resulting in a reduction of recalled cases as shown in a recent report (Warren *et al*, 2005). This will be further complemented by improvements in routine analysis software, together with continuing improvements in hardware allowing more detailed definition of the characteristics of lesions.

To our knowledge, this is one of the first full cost-effectiveness analyses of CE MRI screening of women with high familial risk. One preliminary analysis (Tilanus-Linthorst *et al*, 2000) reported a cost of EUR 13 930 per detected cancer (£9511; US $ 16 716) (1 Ł=€0.6828 EUR; €1=1.2 US $ (06.02.2006)) based on a small sample of 109 women who were screened over a period of 4 years. However, their analysis was based on MRI unit cost of EUR 170 (£116; US $ 204), which is probably an underestimate of the real cost for performing contrast-enhanced breast MRI. In a recently published modelling study, Plevritis *et al.* (2006) estimated that annual screening with mammography and MRI combined would result in two additional life years for women with *BRCA1* mutations and about 18 months for women with *BRCA2*, compared with women screened by mammography alone. The estimated additional cost per extra quality-adjusted life year was between $45 000 (£24 000, €35 000) and $700 000 depending on the age range screened and the type of mutation. When a commonly cited cost-effectiveness threshold of $100 000 per QALY was employed, then only screening with mammography and MRI combined was found to be cost-effective for women with *BRCA1* mutation screened between the ages of 35 and 54.

Several UK studies comparing two view with one view mammography policies (Bryan *et al*, 1995; Wald *et al*, 1995), or a combination of different viewing and reading policies at incident screens (Johnston and Brown, 1999), reported incremental costs per cancer detected of approximately £6000–£10 300 per cancer detected (costs uprated to 2003–2004 prices) in an older population compared to the MARIBS population (Bryan *et al*, 1995; Wald *et al*, 1995; Johnston and Brown, 1999). If these implemented interventions are considered to be an accepted benchmark for cost-effectiveness, then our results suggests that only screening with CE MRI combined with mammography of the *BRCA1/BRCA2* subgroups is likely to be a cost-effective screening policy. However, due to different pathological characteristics of cancers in mutation carriers, different screening intervals and risk levels, comparisons to these programmes should be interpreted with caution.

Our study has several limitations. Firstly, because of the study design and the fact that a recall was initiated on the basis of the results of all diagnostic tests combined, we are unable to explore the relationship between the screening strategy that initiated the recall and the type and number of further investigations. We have found, however, that the frequency of FNA and ultrasound investigations would have been similar in each group; a further mammography would have been more likely in women recalled on the basis of mammography alone because the lesion was evidently visible on mammography and this was the standard and most economic technique. A further CE MRI was more likely in women recalled on the basis of CE MRI either alone or in combination with mammography (Table 4) because the study protocol provided for a follow-up CE MRI in cases where the MRI findings were equivocal and, in some cases, it was the only modality on which the lesion could be observed. Although this makes sense intuitively, it is highly speculative to claim that we would have observed the same pattern if this had been a randomised trial. Nonetheless, we have found that the mean costs for further tests per recalled case were lower in the mammography group compared to the two other screening strategies. In the sensitivity analyses, however, the incremental costs per cancer detected were relatively insensitive to the assumed total costs associated with further tests.

Secondly, we did not include treatment-related costs in our analysis but followed current methodological guidance, which advises analysts to use the same time horizon for both costs and outcomes (Ramsey *et al*, 2005). Nevertheless, since screening will detect more cancers at an earlier stage, which is typically associated with lower treatment costs compared to cancers with a poorer prognostic index (Johnston, 2001), further cost savings are likely to be realised with CE MRI as it is a more sensitive screening modality.

Thirdly, it is not known what monetary value decision makers attach to the intermediate outcome measure ‘cancer detected’ used in our analysis. Therefore, comparisons of the study results are only meaningful with cost-effectiveness studies that also calculated a cost per cancer detected. Since the impact of early detection on mortality and/or health-related quality of life in this population is currently not known, we were not able to estimate life years or QALYs gained (Plevritis, 2000; Dolan, 2001; Taylor *et al*, 2005). We are presently analysing the study data to look at predicted mortality. Findings from this research should be awaited before calculations of a cost per life year gained/QALYs are made.

Taking the commonly cited cost-effectiveness threshold of the National Institute for Clinical Excellence (NICE) of £20 000 per QALY (NICE, 2004; Rawlins and Culyer, 2004) with a grey area between £20 and 40 000, there would only have to be an average gain of 0.65 (0.75) QALY per cancer detected in the *BRCA1* (*BRCA2*) group and 1.4 QALYs for the whole group to demonstrate acceptable cost-effectiveness for CE MRI. It should be borne in mind, however, that this again represents an upper estimate since potentially lower treatment costs associated with screen-detected cancer are not considered in these calculations.

In addition to financial costs, there are significant psychosocial costs associated with MRI breast screening in women at high risk of breast cancer (Anderson and Walker, 2002). In a subgroup of 611 women taking part in MARIBS, we studied acceptability, anxiety, intrusive thoughts and intention to return (Anderson *et al*, 2004). Overall, we found that annual mammography and CE MRI are acceptable forms of screening for the majority of women with a family history of breast cancer; 89% reported that they definitely intended to return and only 1% definitely intended not to return. However, 4% found breast MRI ‘extremely distressing’, and 47% reported still having intrusive thoughts about the examination 6 weeks afterwards. Clearly, the psychological aspects of annual MRM needs to be taken into account in the overall evaluation of an annual screening programme.

There is currently no national policy in the UK regarding screening women at high risk using CE MRI. A recent survey (personal communication) of centres that took part in the MARIBS study has shown that, at present, almost no MRI screening is taking place. This is largely due to a lack of funding and national guidelines. The issue is currently under consideration by the NICE and their findings are expected in October 2006. The MARIBS group are now establishing a working group to make recommendations regarding the screening protocol which would be appropriate to use in a clinical service setting.

In conclusion, we have shown that CE MRI in combination with mammography is a potential cost-effective screening technique for women at high familial risk for breast cancer. Further research is needed to determine whether an early detection results in a measurable increase in quality-adjusted life expectancy. Anticipated technical improvements in MRI technology together with an optimised use in a routine screening setting suggest potentially lower cost for a CE MRI test than calculated in our study setting. Therefore, we would recommend revising the cost-effectiveness estimates presented here as soon as new evidence emerges (Sculpher *et al*, 1997).

## Figures and Tables

**Figure 2:**
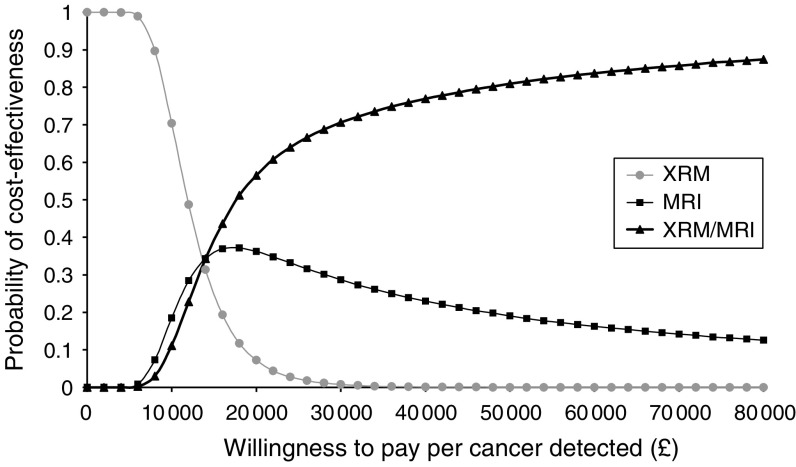
Cost-effectiveness acceptability curve for women with mutation in *BRCA1* or with a first degree relative with mutation in *BRCA1*. Curve shows the probability that a screening modality is cost-effective for a range of decision makers' maximum willingness to pay per cancer detected.

**Figure 3:**
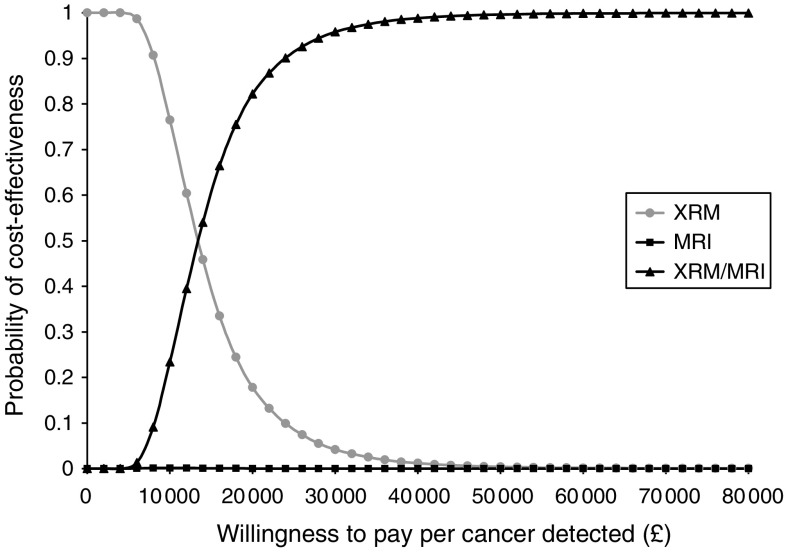
Cost-effectiveness acceptability curve for women with mutation in *BRCA2* or with a first degree relative with mutation in *BRCA2.* Curve shows the probability that a screening modality is cost-effective for a range of decision makers' maximum willingness to pay per cancer detected.

**Table 1:** Unit costs

	**Unit cost**	**Source**
Screening mammography	£33.5	Participating trial centres
Screening breast CE MRI (estimated for routine clinical practice setting)	£249.6	Participating trial centres
Screening breast CE MRI (based on research setting)	£405.1	Participating trial centres
Repeated mammography	£48.6	Participating trial centres
Repeated breast CE MRI (estimated for routine clinical practice setting)	£299.2	Participating trial centres
Ultrasound	£48.8	Participating trial centres
FNA	£130.8	Participating trial centres
Core biopsy	£176	Reference costs J27op
MRI guided biopsy	£955	Participating trial centres
Surgical biopsy	£984	NHS Reference costs J07
Mastectomy	£2058	NHS Reference costs J03 (elective)

CE, contrast enhanced; MRI, contrast enhanced magnetic resonance imaging.

**Table 2:** Breakdown of unit costs for CE MRI screening procedure

**Unit cost item**	**Research CE MRI (mean, range)**	**Estimate for unit costs of CE MRI in a routine clinical practice setting**
*Staff costs*		
Patient preparation	£9.7 (£5.3–£18.9)	£9.7
Taking scan	£29.1 (£15.8–£40.6)	£18,6^a^
Analysis	£69.0 (£34.5–£92.0)	£34.5^b^
Reporting	£11.3 (£3.0–£17.3)	£11.3
Total staff costs per screen	£119.1 (£87.1–£153.2)	£74.1
		
*Equipment and maintenance*		
Equipment	£1 072 951 (£914 755–£1 250 000)	£700 000
Maintenance	£75 800 (£54 000–£106 000)	£75 800
Yearly throughput	2962 (1000–3806)	5000^c^
Total equipment cost per screen^d^	£76.9 (£43.1–154.6)	£32.0
		
*Others*		
Consumables	£32.2 (£12.5–£44.3)	£32.2
Contrast medium	£131.3 (£120.0–£135.4)	£65.6^e^
Overheads/capital charges	£45.8 (£34.9–£61.8)	£45.8
		
Total cost	£405.1 (£320.9–£506.2)	£249.6

a Reducing time for taking scan by 36% (30 min instead of 47 min) with the same staff configuration.

b Reducing time for analysing scan by 50% (15 min instead of 30 min) with the same staff configuration.

c 10 h running time and 30 min examination time.

d Equipment costs were discounted at 3.5% (assuming 10 years life span).

e Cost of contrast medium reduced by 50% (generic formulation and/or lower dose (0.1 mmol kg^−1^ body weight)); CE-MRI, contrast enhanced magnetic resonance imaging.

**Table 3:** Number of recalls in study and analysis

	**Study results^a^**			**Hypothetical scenario used in the cost-effectiveness study- recalls on the basis of CE MRI alone or mammography alone^b^**		
*All recalled women (N*=*279)*^c^						
	XRM−	XRM+	Total	XRM−	XRM+	Total
CE MRI−	40 (2)	37 (6)	77 (8)	40 (2)	37 (6)	77 (8)
CE MRI+	165 (19)	37 (8)	202 (27)	185 (20)	17 (7)	202 (27)
Total	205 (21)	74 (14)	**279 (35)**	225 (22)	54 (13)	**279 (35)**
						
*Only BRCA 1 group* (*N*=*75)*^d^						
CE MRI−	11 (1)	8 (0)	19 (1)	11 (1)	8 (0)	19 (1)
CE MRI+	44 (9)	12 (3)	56 (12)	52 (9)	4 (3)	56 (12)
Total	55 (10)	20 (3)	**75 (13)**	63 (10)	12 (3)	**75 (13)**
						
*Only BRCA 2 group* (*N*=*42)*^e^						
CE MRI−	7 (1)	8 (4)	15 (5)	7 (1)	8 (4)	15 (5)
CE MRI+	23 (5)	4 (2)	27 (7)	24 (5)	3 (2)	27 (7)
Total	30 (6)	12 (6)	**42 (12)**	31 (6)	11 (6)	**42 (12)**

Cancer in parentheses; patient numbers refer to recalled cases only.

a Study results refer to the result of the clinical study (Leach *et al*, 2005).

b For underlying assumptions see Materials and Methods section (number of recalls).

c Out of 649 women enrolled in the study.

d Out of 139 women in the *BRCA1* group.

e Out of 86 women in the *BRCA2* group.

CE MRI, contrast enhanced magnetic resonance imaging; XRM, X-ray mammography.

**Table 4:** Number of further procedures when recalled (all women)

	**Recall generated by**		
**Procedure**	**Mammography (*N*=54), *n* (%)**	**CE MRI (*N*=202), *n* (%)**	**Mammography with CE MRI (*N*=239), *n* (%)**
Further XRM	23 (43)	21 (10)	39 (19)
Further MRI	7^a^ (13)	135 (67)	137 (57)
Ultrasound	38 (70)	148 (73)	173 (72)
FNA	11 (20)	56 (28)	60 (25)
Core biopsy	17 (31)	30 (15)	38 (16)
MR-guided biopsy	—	13 (6)	13 (5)
Surgical biopsy	8 (15)	15 (7)	19 (8)
Mastectomy	—	1 (0.5)	1 (0.4)

a These seven further MRI studies were not considered for calculation of costs attributable to recall procedures.

CE MRI, contrast enhanced magnetic resonance imaging; XRM, X-ray mammography.

**Table 5:** Outcomes and costs per patient screened

	**Mammography**	**CE MRI**	**Mammography with CE MRI**
Cost related to screening	£33.5	£249.6	£283.1
Recall rate	2.9%^a^	10.7%	12.7%
Cost of further investigations when recalled	£282.8 (379.7)	£506.4 (399.4)	£399.5 (413.4)
Cost of further investigations per screen	£8.1 (79.3)	£54.4 (204.1)	£59.3 (213.2)
*Total cost per screen*	£41.7 (79.3)	£304.0 (204.1)	£342.4 (213.2)
Number of cancer detected per screen	0.00691	0.01435	0.01754
*Cost per additional cancer detected*	—	Dominated^b^	£28284
*Total cost per screen* (*BRCA1 only*)	£43.7 (89.8)	£323.0 (230.0)	£361.2 (236.5)
Number of *BRCA1* cancer detected	0.00794	0.03174	0.03174
*Cost per additional cancer detected***-** *BRCA1 only*	—	£11731	—
*Total cost per screen* (*BRCA2 only*)	£55.7 (149.7)	£317.5 (249.3)	£369.3 (290.1)
Number of *BRCA2* cancer detected	0.02459	0.02869	0.04508
*Cost per additional cancer detected***-** *BRCA2 only*	—	Dominated^b^	£15302

a Based on assumptions outlined in Materials and Methods section.

b Extended dominance (incremental cost-effectiveness ratio is higher than for next, more effective alternative).

( )=s.d.; CE MRI, contrast enhanced magnetic resonance imaging.

**Table 6:** Results of sensitivity analyses

	**Incremental cost-effectiveness ratios**	
	**CE MRI**	**Mammography with CE MRI**
*Only prevalent screen*		
All	Dominated^a^	£22 388
BRCA1 only	£6489	—
BRCA2 only	—	£14 366
		
*All assessment costs*		
Increased by 50%	Dominated^a^	£30 687
Decreased by 50%	Dominated^a^	£25 880
		
*Mammography assessment costs*		
Assumed to be the same as for CE MRI (£71.67)	Dominated^a^	£23 933
		
*Alternative unit cost estimate CE MRI (based on research setting)*		
All	Dominated^a^	£44 564
BRCA1 only	£19 068	—
BRCA2 only	Dominated^a^	£22 890

a Extended dominance (incremental cost-effectiveness ratio is higher than for next, more effective alternative).

CE MRI, contrast enhanced magnetic resonance imaging.

**Box 1:** Summary of assumptions regarding recall

	**X-ray mammography**		**CE MRI**		
**Scenario**	**Reader 1**	**Reader 2**	**Reader 1**	**Reader 2**	**Recall**
*Recalled on the basis of X-ray mammography alone?*					
1	Abnormal	Abnormal	—	—	Yes
2	Normal	Abnormal	Normal	Normal	Yes
3	Abnormal	Normal	Normal	Normal	Yes
4	Abnormal	Normal	Abnormal	Normal	? (→Third reader)
5	Abnormal	Normal	Normal	Abnormal	? (→Third reader)
6	Normal	Abnormal	Abnormal	Normal	? (→Third reader)
7	Normal	Abnormal	Normal	Abnormal	? (→Third reader)
*Recalled on the basis of CE MRI alone*?					
8	—	—	Abnormal	Normal	Yes
9	—	—	Normal	Abnormal	Yes
10	—	—	Abnormal	Abnormal	Yes

## Appendix

Centres and staff who contributed to the MARIBS Study are listed below:

*Study Staff (past and present)*

LJ Pointon (Study Coordinator)

RJC Hoff (Assistant Study Coordinator)

K Chan (Data Manager)

M Khazen (Image Analysis Physicist)

RML Warren (Study Radiologist)

J Anderson (Health Psychologist)

C Levesley (Psychology Research Assistant)

I Griebsch (Health Economist)

D Thompson (Statistician)

C Hayes (Study Physicist)

R Gregory (Study Physicist)

M Sydenham (Acting Study Coordinator)

K Bletcher (Data Manager)

GP Liney (Study Physicist)

B Browne (Data Manager)

*Data Monitoring and Ethics Committee*

K McPherson (Chairman, Visiting Professor of Public Health Epidemiology)

R Blamey (Professor Emeritus and Consultant Breast Surgeon)

SW Duffy (Professor of Cancer Screening).

*Trial Steering Committee*

A Howell (Chairman, Professor of Medical Oncology)

D Easton (Study Statistician, Genetic Epidemiologist)

DG Evans (Study Representative, Consultant Geneticist)

JE Husband (Host Institution Representative, Professor of Radiology)

E Maher (Independent Member, Professor of Medical Genetics)

MJ Michell (Independent Member, Consultant Radiologist)

RML Warren (Study Radiologist, Consultant Radiologist)

W Watson (Consumer Representative, Founder of the Hereditary Breast Cancer Group).

*Recruiting centres (Number of women recruited)*

Aberdeen: NE Haites, B Gibbons, H Gregory, M McJannett, L McLennan (29); Belfast: PJ Morrison, L Jeffers (12);

Birmingham: T Cole, L Burgess, CmcKeown, JEV Morton (24);

Bristol Royal Infirmary: Z Rayter (3);

Cambridge: J Mackay, J Rankin, LG Bobrow, S Downing, S Everest, A Middleton, B Newcombe (67);

Dundee: D Goudie, D Young (24);

Edinburgh: M Steel, EDC Anderson, J Campbell, JM Dixon, P Walsh (60);

Frenchay Hospital

Bristol: SJ Cawthorn, M Shere, C Dawe (29);

Glasgow: R Davidson, CM Watt (20);

Guy's and St Thomas' London: SV Hodgson, S Watts (43);

Leeds: C Chu, G Turner, E Hazell, L Rae (55);

Liverpool: I Ellis, J Birch, C Holcombe, S Holcombe, K Makinson (16);

Manchester Regional Genetics Service: DG Evans, G Hall, A Shenton (157);

Newcastle: F Douglas, G Seymour (111);

Northwick Park: J Paterson, C Cummings, L Jackson (9);

Sheffield: OWJ Quarrell, JA Cook, D Kumar (14);

Southampton: DM Eccles, G Crawford, S Goodman (34);

Sutton and St George's (or collaborators who referred to this centre): RA Eeles, S Allan, A Ardern-Jones, E Bancroft, C Brewer, R Carpenter, C Chapman, DL Christensen, RC Coombes, S Ebbs, I Fentiman, S Furnell, R Given-Wilson, S Goff, S Gray, M Greenall, G Gui, T Homfray, R Houlston, MW Kissin, I Laidlaw, F Lennard, I Locke, AM Lucassen, F McDuff, K McReynolds, G Mitchell, MWE Morgan, V Murday, U Querci della Rovere, N Rahman, N Sacks, A Salmon, S Shanley, S Shrotria, N Sodha, A Stacey-Clear, C Webster (130).

*Magnetic resonance image readers (number of cases read)*

Aberdeen: FJ Gilbert (132), G Needham (75);

Barnet: GR Kaplan (19);

Belfast: JG Crothers (13);

Birmingham: CP Walker (48);

Bristol Royal Infirmary: A Jones (10);

Cambridge: PD Britton (161), AK Dixon (104), R Sinnatamby (25), RML Warren (759);

Dundee: JM Rehman (14), D Sheppard (20);

Edinburgh: J Walsh (426);

Frenchay Hospital Bristol: ID Lyburn (23), NF Slack (50);

Glasgow: LM Wilkinson (24);

Guy's and St Thomas' London: S Rankin (222);

Hillingdon Hospital Middlesex: K Raza (100);

Hull: G Hall (81), P Balan (47), LW Turnbull (221);

Liverpool: GH Whitehouse (47);

Manchester-Christie Hospital/Nightingale Centre: CRM Boggis (80), E Hurley (16), A Jain (4), S Reaney (49), M Wilson (63);

Manchester Medical School: JM Hawnaur (183), J Jenkins (4);

Newcastle: A Coulthard (234), AJ Potterton (321);

Northwick Park: B Shah (57), W Teh (92);

Paul Strickland Scanner Centre London: AR Padhani (269);

Royal Hospital Haslar Gosport: PJ Buxton (2), JM Domjan (2), PAL Gordon (6); Southampton: M Briley (55), C Rubin (72);

Sutton and St George's: P Kessar (now at Bromley Hospitals NHS Trust; 256); University College Hospital: MA Hall-Craggs (23).

*Mammography Readers (number of films read)*

Aberdeen: HE Deans (42), K Duncan (47), L Gomersall (30), G Iyengar (3), G Needham (8);

Barnet: GR Kaplan (4); Belfast: JG Crothers (12), J McAllister (12), JM Kirby (1);

Birmingham: S Bradley (47), MG Wallis (45);

Bristol Royal In.rmary: JE Basten (56), E Kutt (52);

Cambridge: PD Britton (185), R Davies (5), CDR Flower (9), AH Freeman (240), D O'Driscoll (4), R Sinnatamby (310), RML Warren (426);

Dundee: AM Cook (25), CM Walker (25);

Edinburgh: A Buttimer (55), A Gilchrist (35), BB Muir (106), J Murray (126), L Smart (10), M Smith (8);

Glasgow: C Cordiner (18), J Litherland (16);

Guy's and St Thomas' London: A Jones (51), S McWilliams (76);

Hull: AE Hubbard (146);

Liverpool: A Ap-Thomas (1), DA Ritchie (33), F White (32);

Manchester-Christie Hospital/Nightingale Centre: DL Asbury (46), U Beetles (14), CRM Boggis (212), R Dobrashian (3), MDJ Harake (15), E Hurley (34), A Jain (20), S Reaney (74), M Wilson (117);

Newcastle: B Kaye (55), M McElroy (180), L McLean (145), W Wotherspoon (230);

Northwick Park: G Markham (8);

Southampton: A Bisset (2), S Hegarty (57), G Michaels (59), N Robson (2);

Sutton and St George's: G Brown (41), J Husband (6), KT Khaw (1), D MacVicar (10), E Moskovic (7), J Murfitt (23).

*Other radiology/magnetic resonance staff*

Aberdeen: ML Muirhead, TW Redpath, S Semple;

Barnet: M Cunningham, S Turnell;

Belfast: Creynolds, R Bridcut, J Winder;

Birmingham: P Fergusson, Z Vegnuti;

Bristol Royal Infirmary: S Cowley, K Isaacs, P Richardson;

Cambridge: J Green, I Joubert, J Pinney, C Pittock, E van Rooyen;

Dundee: SJ Gandy, P Martin, T McLeay;

Edinburgh: T Lawton, I Marshall, L Thomson;

Frenchay Hospital Bristol: H Albarran, V Blake, J Robson;

Glasgow: M Cockburn;

Guy's and St Thomas' London: J Goodey, K McBride;

Hull: D Fagge, S Hunter, G Liney;

Liverpool: J Chance, J Davies, Z Hussain;

Manchester–Christie Hospital/Nightingale Centre: Chammond, W Johnson; Manchester Medical School: JE Adams, Y Watson;

Newcastle: L Lewis, M Myers;

Northwick Park: D Fox, J Johnson, J Shah;

Paul Strickland Scanner Centre, London: L Culver, R Sale, JJ Stirling, NJ Taylor;

Royal Hospital Haslar, Gosport: E Boyd, J Evans, W Johnston, S Lindsay, R MacKenzie, H Stansby, B Tailor, L Watts, L Womack

Southampton: A Darekar, S King, N Shepherd;

Sutton and St George's: G Charles-Edwards, E Charles-Edwards, E Scurr (on behalf of all the MRI radiographers Sutton).
